# Serum mitochondrial-encoded NADH dehydrogenase 6 and Annexin A1 as novel biomarkers for mortality prediction in critically ill patients with sepsis

**DOI:** 10.3389/fimmu.2024.1486322

**Published:** 2024-11-14

**Authors:** Fan Zhou, Meiling Chen, Yilin Liu, Xianzhu Xia, Pingsen Zhao

**Affiliations:** ^1^ Department of Laboratory Medicine, Yuebei People’s Hospital Affiliated to Shantou University Medical College, Shaoguan, China; ^2^ Laboratory for Diagnosis of Clinical Microbiology and Infection, Yuebei People’s Hospital Affiliated to Shantou University Medical College, Shaoguan, China; ^3^ Research Center for Interdisciplinary & High-Quality Innovative Development in Laboratory Medicine, Yuebei People’s Hospital Affiliated to Shantou University Medical College, Shaoguan, China; ^4^ Shaoguan Municipal Quality Control Center for Laboratory Medicine, Yuebei People’s Hospital Affiliated to Shantou University Medical College, Shaoguan, China; ^5^ Shaoguan Municipal Quality Control Center for Surveillance of Bacterial Resistance, Shaoguan, China; ^6^ Shaoguan Engineering Research Center for Research and Development of Molecular and Cellular Technology in Rapid Diagnosis of Infectious Diseases and Cancer, Shaoguan, China; ^7^ Intensive Care Medicine Department, Yuebei People’s Hospital Affiliated to Shantou University Medical College, Shaoguan, China

**Keywords:** sepsis, MT-ND6, ANXA1, diagnosis, prognosis, immunological classification

## Abstract

**Objectives:**

Formyl peptide receptor 1 (FPR1) is a member of G protein-coupled receptor (GPCR) family that detects potentially danger signals characterized by the appearance of N-formylated peptides which originate from either bacteria or host mitochondria during organ injury, including sepsis. Mitochondrial-encoded NADH dehydrogenase 6 (MT-ND6) and Annexin A1 (ANXA1), as mitochondrial damage-associated molecular patterns (mtDAMPs) agonist and endogenous agonist of FPR1 respectively, interact with FPR1 regulating polymorphonuclear leukocytes (PMNs) function and inflammatory response during sepsis. However, there is no direct evidence of MT-ND6 or ANXA1 in the circulation of patients with sepsis and their potential role in clinical significance, including diagnosis and mortality prediction during sepsis.

**Methods:**

A prospective cohort study was conducted in ICU within a large academic hospital. We measured serum MT-ND6 or ANXA1 in a cohort of patients with sepsis in ICU (n=180) and patients with non-sepsis in ICU (n=60) by Enzyme-linked immunosorbent assays (ELISA). The ROC curve and Kaplan Meier analysis was used to evaluate the diagnostic and prognostic ability of two biomarkers for patients with sepsis.

**Results:**

The concentration of MT-ND6 and ANXA1 were significantly elevated in the patients with sepsis, and the diagnostic values of MT-ND6 (0.789) for sepsis patients was second only to SOFA scores (AUC = 0.870). Higher serum concentrations of MT-ND6 (>1.41 ng/ml) and lower concentrations of ANXA1 (< 8.09 ng/mL) were closely related to the higher mortality in patients with sepsis, with the predictive values were 0.705 and 0.694, respectively. When patients with sepsis classified based on four pro-inflammation and two anti-inflammation cytokines, it was shown that combination of MT-ND6 and ANXA1 obviously improved the predictive values in the septic patients with mixed hyperinflammation or immunosuppression phenotypes.

**Conclusion:**

Our findings provide valuable models testing patient risk prediction and strengthen the evidence for agonists of FPR1, MT-ND6 and ANXA1, as novel biomarker for patient selection for novel therapeutic agents to target mtDAMPs and regulator of GPCRs in sepsis.

## Background

Sepsis, often defined as a an multiorgan dysfunction due to the host’s dysregulated response to infection, with a high morbidity and mortality (1–3). According to the recent study, there are 47-50 million new cases of sepsis worldwide each year, and the number of sepsis-related deaths exceeds 11 million, imposing serious burden on public health (4, 5). The *Surviving Sepsis Campaign* highlights the significance of early therapeutic interventions and increased screening rates for high-risk individuals as a means of reducing the high fatality rate (6). However, the diagnosing early-stage sepsis and determining individuals who are at high risk for mortality are still difficult due to the complex pathophysiology and heterogeneity of sepsis patients (6, 7).

Disbalances of the immune response play an important role in the pathophysiology of sepsis. Patients may develop simultaneously or concomitantly states of systemic or local hyperinflammation and immunosuppression, two normally opposing responses that involve distinct cell types and organ systems (8). Increasing evidence suggests that stratification of the heterogeneous population of septic patients with consideration of their host response might led to treatments that are more effective (8). However, we have neither a definition nor widely accepted diagnostic test(s) for these dysregulated immune responses yet, despite the availability of a plethora of biomarkers. Fortunately, several reports published recently suggested that patients with critically illness could be immunologically stratified according to the expression of pro-inflammation and anti-inflammation cytokines, which coincides with our idea and also provides solid supports for the following studies (9, 10).

At present, procalcitonin (PCT), C-reactive protein (CRP) and peripheral blood white blood cell count (WBC) are the most widely used laboratory parameters to reflect the disease progression and severity of patients with critically ills (3, 11, 12). However, none of them are commonly used to reflect the immune states of sepsis patients, as well as to predict their mortality. These limitations drive the urgent needs for exploring the novel specific and robust biomarkers to help the clinicians take more effective and accurate treatments.

Formyl peptides receptors (FPRs) is a member of G protein-coupled receptor superfamily (GPCRs), which mainly expresses in the myeloid cells like neutrophils (PMN), macrophages, and monocytes. FPRs are responsible for regulating the migration, aggregation and activation of inflammatory cells (13, 14). In our previous studies, we found that the expression of FPR1 was significantly increased in the peripheral blood mononuclear cells (PBMCs) in almost all severe patients with COVID-19 when compared with mild patients (15, 16). FPR1 displays a large array of exogenous and endogenous ligands, including the N- formyl peptides (NFPs), annexin A1 (ANXA1), heat shock protein (HSP), and cathepsin G, etc. Naturally, the NFPs are mainly produced by the degradation of mitochondrial proteins following the pathogens and tissue cells death (13, 17). Mitochondria contain over 1000 proteins but only 13 of them are formylated, among these peptides, the nicotinamide adenine dinucleotide dehydrogenase subunit-6 (ND6) are one of the most potent human mtFP (14, 18), as it could promote PMN migration and degranulation by initiating cytosolic calcium ([Ca2+]i) signaling in a FPR1-dependent manner, which play an important role in the activation of innate immune responses (19–21).

Annexin A1 (ANXA1) is a key endogenous glucocorticoid protein that regulates inflammation via FPR1 signaling pathway (22, 23). It is normally present in the cytoplasm of epithelial and endothelial cells, as well as the myeloid cells (24, 25). Inflammation and exogenous glucocorticoids can induce the release of ANXA1 into the blood, which play a crucial part in inhibiting the generation of inflammatory factors, and limiting inflammatory cell activation and the duration of the inflammatory reaction (26, 27).

Although these two molecules have been extensively studied in inflammatory diseases such as tumors, trauma, cerebral ischemia-reperfusion injury, and diabetic nephropathy (28–31), little has been reported in sepsis. In the present study, we try to explore the diagnostic and prognostic values of MT-ND6 and ANXA1 in patients with sepsis, and to explore the potential role of these two molecules in people with different immune states, aiming at providing assistance for subsequent immunomodulatory.

## Materials and methods

### Study population and parameters

**Figure 1:**
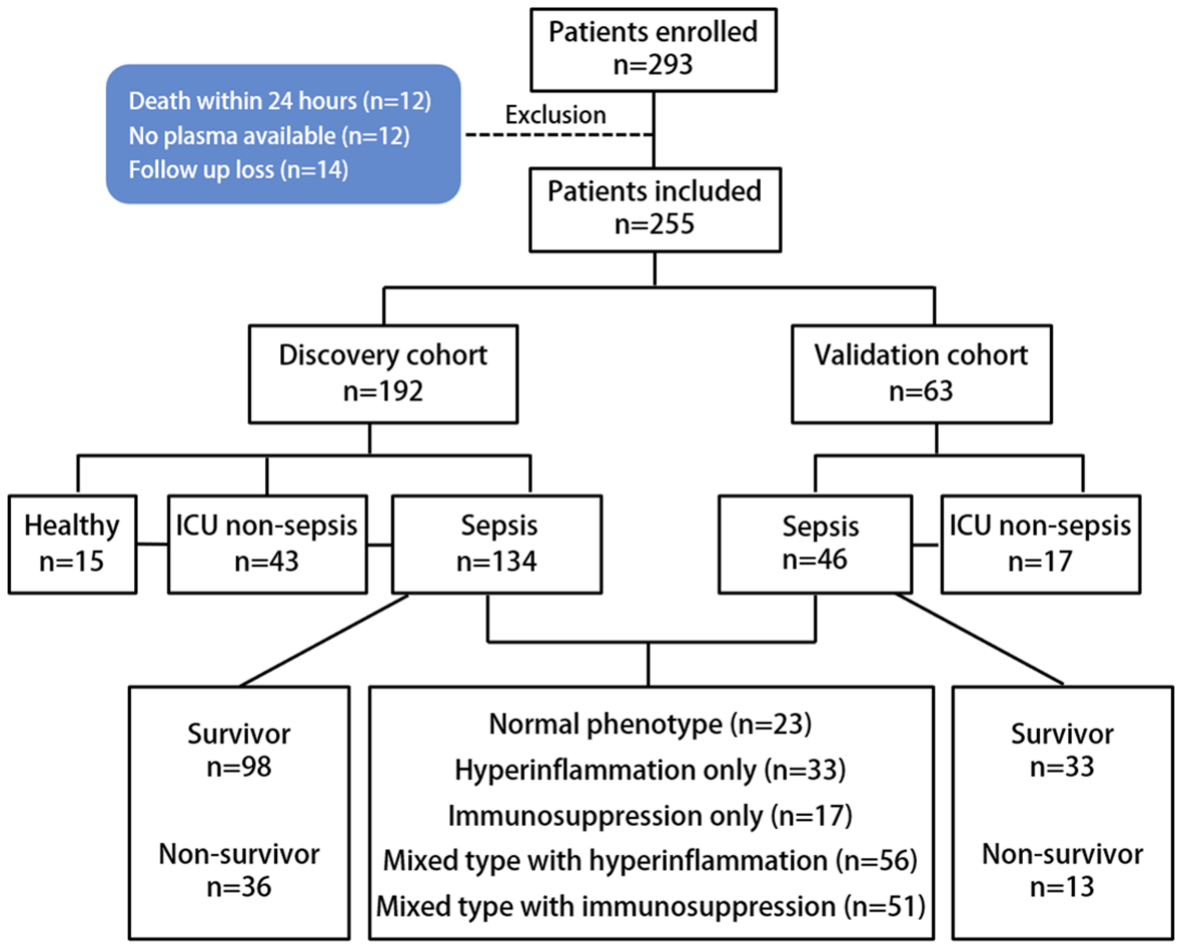
Patient flowchart.

We used two independent cohorts to validate the diagnostic and prognostic values of ANXA1 and MT-ND6 in patients with severe infections requiring ICU admission. We then used these cohorts to further develop and validate a sepsis score. Discovery cohort: 146 adult patients were prospectively enrolled between September 2022 and March 2023 in Yuebei People’s Hospital at the time of ICU admission. Validation cohort: another 46 adult patients were prospectively enrolled in the same intensive care unit between March 2023 and October 2023. Patients aged ≥ 18 years were included within 24 h of their ICU admission for sepsis or septic shock. Additionally, a total of 60 age- and sex- matched ICU non-sepsis patients in critical conditions (including 6 cases of burns, 10 cases of shock, 22 cases of road traffic injury, 13 cases of disturbance of consciousness, 5 cases of falls, 4 cases of major surgery) and 15 healthy volunteers were recruited as the ICU non-sepsis patient control group and healthy control group, respectively. After 30 days of follow-up, patients’ survival status was recorded during the ICU period until discharge or death. The flowchart of the study population was shown in **Figure 1**. The collected clinical parameters included peripheral WBC, platelet count, PCT, CRP, SOFA score, blood culture, bacterial culture, and 30-day survival status.

Sepsis patients with inclusion criteria: (1) Meets the diagnostic criteria for sepsis 3, which include infection or a high suspicion of infection and a SOFA score ≥ 2 (32); (2) Age ≥ 18 years old; (3) All patients’ clinical records should be complete. In addition to achieving the above diagnostic criteria, patients with septic shock have to meet the following: (1) Persistent hypotension needing vasoactive medications to sustain a mean arterial pressure above 65 mmHg; or (2) Blood lactate level more than 2 mmol/L in the absence of hypotension.

Sepsis patients with Exclusion criteria: (1) Patients readmitted to the ICU; (2) Patients with comorbidity of severe liver, kidney malfunction, heart failure, cancer, AIDS, or severe blood diseases; (3) Pregnant and postpartum patients; (4) Patients with autoimmune diseases who get treated with immunosuppressants or glucocorticoids; (5) Deaths within 24 hours of enrollment.

This study has been approved by the Clinical Research Ethics Committee of Yuebei People’s Hospital Affiliated to Shantou University Medical College (registration number: YBEC-KY (2021)-110), and all patients or their legal representatives have signed informed consent.

### Sample measurement

For all selected populations, the WBC (BC-5800 automatic blood cell counter), PCT (Roche Cobas E801), CRP (Roche Cobas 8000), and other indicators were detected on the day of enrollment. An additional venous blood sample was collected simultaneously from the patient, and then the serum was isolated and frozen at -80℃ until ANXA1 (Novus, 042422303), MT-ND6 (My BioSource, E03141637), HBP (Novus, 073192303), IL-6 (R&D P376851), IL-1β (R&D, P370960), IFN-α (R&D, 368847), TNF-α (R&D, P367646), IL-4 (R&D, P396413) and IL-10 (R&D, P406798) were detected using an enzyme-linked immunosorbent assay (ELISA).

### Statistical analysis

IBM SPSS Statistics 26.0 (IBM, Armonk, New York, US) software was employed to carry out the statistical analysis. Since data from the biomarkers were not normally distributed, primarily non-parametric tests were performed. Data were represented as the mean ± standard deviation or median and interquartile range (IQR). Continuous variables were compared using Student’s t-test or the Mann-Whitney U test. Categorical variables were compared using the chi-square test or Fisher’s exact test. One-way analysis of variance (ANOVA) or Kruskal-Walli’s test was used to compare more than two groups of quantitative data. To estimate the value of MT-ND6 and ANXA1 for predicting 30-day mortality in patients with sepsis, the receiver operating characteristics (ROC) curves were constructed and the area under the curve (AUC) was determined by its 95% confidence interval (95% CI). Cumulative 30-day mortality was analyzed using Kaplan–Meier survival analysis with the log-rank *post hoc* test. The optimal cut-off points were calculated by the Youden index, and the weighted combination of sensitivity (SE) and specificity (SP) were shown in tables. *P* values less than 0.05 were considered statistically significant, and the significance levels quoted are two-sided.

## Results

### Baseline characteristics

There were 134 adult septic patients, 43 ICU non-sepsis patients and 15 healthy control volunteers that were enrolled in the discovery cohort. The basic demographics and clinical characteristics of these patients are summarized in **Table 1**. Overall, all of the subjects had comparable gender distributions, but patients with sepsis and septic shock tended to be older than ICU non-sepsis patients and healthy controls. Septic shock patients exhibited a higher SOFA score than the sepsis group (8.0 vs. 5.0, *P* < 0.001), as well as the 30-day mortality.

**Table 1:** Patient characteristics.

Variables	Discovery cohort					Validation cohort			
	Healthy control (n=15)	ICU non-sepsis (n=43)	Sepsis (n=81)	Septic shock (n=53)	*p* value	ICU non-sepsis (n=17)	Sepsis (n=25)	Septic shock (n=21)	*p* value
Age, median (IQR)	42.0 (29.0)	54.0 (21.0)	61.0 (21.0) ^ab^	67.0 (16.0) ^ab^	<0.001	48.0 (15.0)	62.0 (14.0) ^b^	63.5 (30.0) ^b^	<0.001
Sex, male (%)	7 (46.7)	23 (53.5)	49 (60.5)	33 (62.3)	0.626	14 (82.4)	15 (57.7)	11 (55.0)	0.165
SOFA scores, median (IQR)	–	2.0 (3.0)	5.0 (2.0) ^b^	8.0 (3.0) ^bc^	<0.001	1.0 (3.0)	4.5 (4.0) ^b^	6.5 (3.0) ^b^	<0.001
Laboratory values, median (IQR)									
PCT (ng/mL)	0.1 (0.1)	0.4 (1.8) ^a^	4.5 (20.2) ^ab^	30.6 (82.5) ^abc^	<0.001	4.1 (9.5)	19.0 (59.3) ^b^	16.4 (50.5) ^b^	<0.005
CRP (mg/dL)	0.1 (0.2)	2.4 (7.2) ^a^	14.9 (29.8) ^ab^	23.6 (33.7) ^ab^	<0.001	8.9 (15.8)	19.0 (33.4)	13.8 (25.6)	0.240
WBC (x 10^9^/L)	6.9 (1.8)	14.8 (8.1) ^a^	14.4 (9.8) ^a^	12.6 (11.8) ^a^	<0.001	10.3 (1.8)	15.3 (9.2) ^b^	11.1 (12.3)	<0.05
IL-6 (ng/mL)	0.1 (0.1)	0.3 (0.7)	0.3 (1.9)	0.6 (4.9) ^ab^	<0.001	0.3 (0.4)	0.4 (0.4)	0.7 (3.7) ^b^	0.061
HBP (ng/mL)	1.2 (1.1)	29.4 (25.1) ^a^	21.6 (25.0) ^a^	24.8 (21.3) ^a^	<0.001	5.7 (1.4)	10.4 (8.4) ^b^	11.8 (6.7) ^b^	<0.001
MT-ND6 (ng/mL)	0.2 (0.1)	0.6 (0.9) ^a^	1.5 (1.9) ^ab^	2.5 (3.1) ^abc^	<0.001	0.5 (0.7)	1.5 (1.3) ^b^	1.7 (1.9) ^b^	<0.001
ANXA1	0.7 (1.2)	4.7 (7.9) ^a^	3.8 (6.6) ^a^	3.4 (4.0) ^a^	<0.001	2.1 (2.8)	6.8 (11.3) ^b^	4.8 (8.2)	<0.001
Comorbidities, n (%)									
Cardiovascular disease	–	9 (20.9)	21 (26.6)	21 (39.6)	0.107	1 (5.9)	10 (38.5)	6 (30.0)	0.059
Hypertension	-	11 (25.6)	28 (34.6)	17 (32.1)	0.590	1 (5.9)	9 (34.6)	8 (40.0)	0.050
Diabetes	–	11 (25.6)	28 (34.6)	18 (34.0)	0.564	3 (17.6)	8 (30.8)	3 (15.0)	0.385
COPD	-	2 (4.7)	11 (13.6)	5 (9.4)	0.287	0 (0.0)	2 (7.7)	2 (10.0)	0.432
Chronic kidney disease	–	2 (4.7)	19 (23.5) ^b^	5 (9.4)	<0.05	0 (0.0)	5 (19.2)	5 (25.0)	0.097
Liver disease	–	4 (9.3)	22 (27.2)	15 (28.3)	0.052	0 (0.0)	5 (19.2)	3 (15.0)	0.168
Source of infection, n (%)									
Respiratory tract	–	–	28 (34.6)	33 (62.3)	<0.005	–	7 (26.9)	5 (25.0)	0.883
Urogenital	-	-	22 (27.2)	12 (22.6)	0.557	-	8 (30.8)	4 (20.0)	0.410
Abdominal	–	–	22 (27.2)	18 (34.0)	0.400	–	6 (23.1)	4 (20.0)	1.000
Skin or soft tissue	-	-	5 (6.2)	2 (3.8)	0.542	-	1 (3.8)	0 (0.0)	1.000
Surgical site	–	–	0 (0.0)	0 (0.0)	–	–	0 (0.0)	1 (5.0)	0.435
Blood culture positive	–	–	37 (45.7)	26 (49.1)	0.702	–	9 (34.6)	13 (65.0) ^c^	<0.05
Cultures, n (%)									
Gram positive	–	–	20 (24.7)	24 (45.3)	<0.05	–	6 (23.1)	11 (55.0) ^c^	<0.05
Gram negative	-	-	56 (69.1)	31 (58.5)	0.207	-	13 (50.0)	10 (50.0)	1.000
Fungus	–	–	8 (9.9)	19 (35.8)	<0.001	–	6 (23.1)	0 (0.0) ^c^	<0.05
Multiple findings	-	-	19 (23.5)	27 (50.9)	<0.001	-	5 (19.2)	4 (20.0)	1.000
Negative cultures	–	–	6 (7.4)	3 (5.8)	0.714	–	3 (11.5)	2 (10.0)	1.000
Outcome, n (%)									
30-day-mortality	–	6 (14.0)	9 (11.1)	27 (50.9) ^bc^	<0.05	1 (5.9)	3 (11.5)	9 (45.0) ^bc^	<0.005

Significant differences between the variables were tested using Kruskal-Wallis (KW) test followed by Dunn’s *post hoc* test or Mann-Whitney U test (MW) or Chi-square test. *P* ≤ 0.05 were considered statistically significant.

^a^compared with control group, *P*<0.05; ^b^compared with ICU non-sepsis group, *P*<0.05; ^c^compared with sepsis group, *P <*0.05.

**Figure 2:**
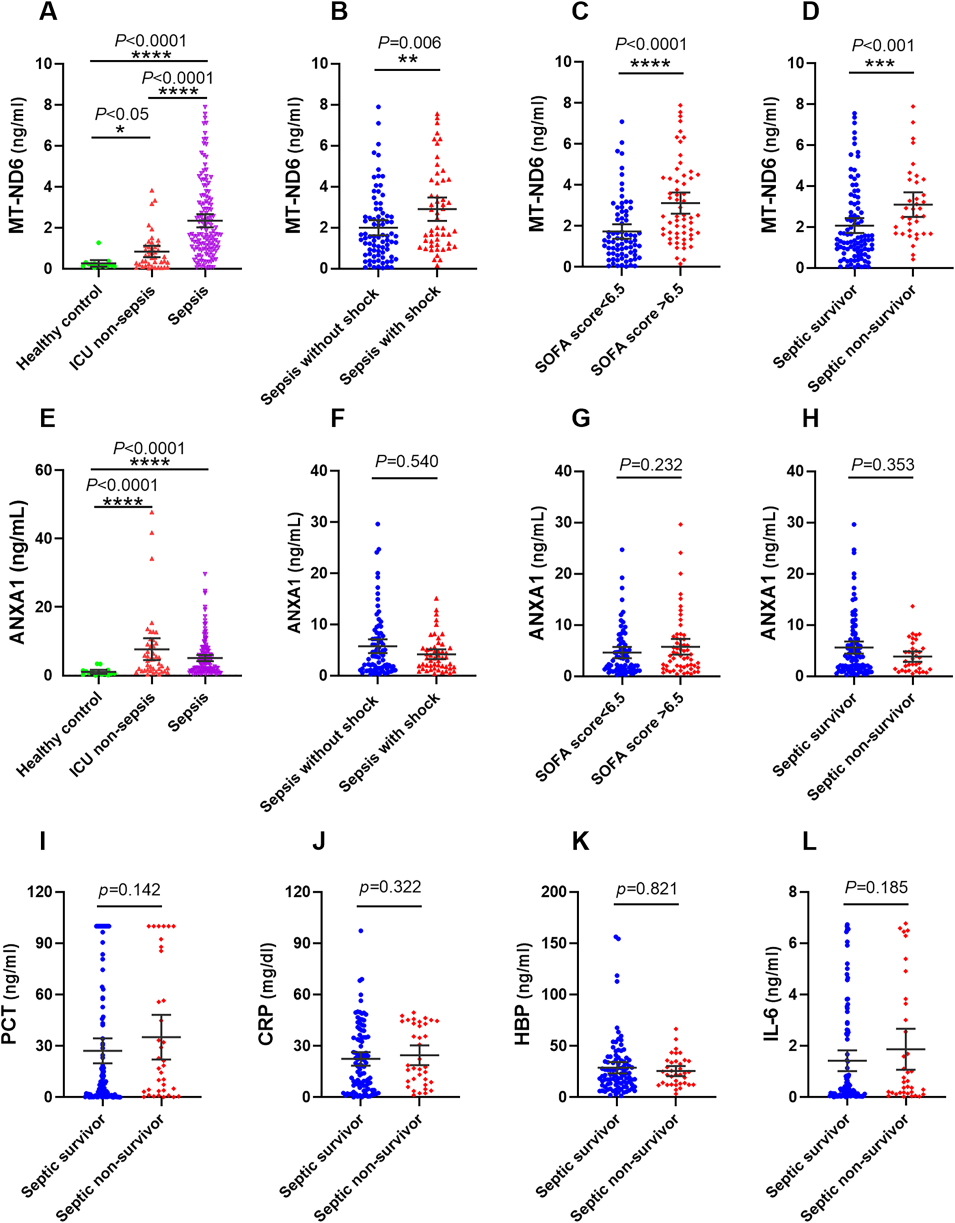
The serum concentration of MT-ND6 and ANXA1 at admission were elevated in the discovery cohort of patients with sepsis. **(A, E)** The serum concentrations of MT-ND6 and ANXA1 were detected from 134 patients with sepsis, 43 non-sepsis patients and 15 healthy controls. MT-ND6 and ANXA1 concentrations were grouped by the sepsis patients whether in shock **(B, F)**, the SOFA cut-off values **(C, G)** and survival states **(D, H)**. **(I–L)** The serum levels of PCT, CRP, HBP, and IL-6 were collected from non-survivor and survivor in patients with sepsis. *P* ≤ 0.05 were considered statistically significant. ∗ denotes *P*< 0.05, ∗∗ denotes *P*< 0.01, ∗∗∗ denotes *P*< 0.001, ∗∗∗∗ denotes *P*< 0.0001 (Kruskal-Wallis and Mann–Whitney U test).

On the day of ICU admission, the serum MT-ND6 and ANXA1 levels of septic patients was significantly elevated than the ICU non-sepsis patients and healthy controls (**Figures 2A, E**). Additionally, the concentration of MT-ND6 was increased with the severity of sepsis patients and SOFA score values (**Figures 2B, C**), but no statistical difference was observed in the levels of ANXA1 (**Figures 2F, G**). We have noticed that the septic shock patients had a higher rate of infection by Gram-positive bacteria and fungus than the sepsis patients (45.3% vs. 24.7%, *P* < 0.05), and we tried to compare the concentration of parameters (including MT-ND6 and ANXA1) in patients with different bacterial culture results, but no statistically significant difference was observed in the both cohort (Data not shown).

### Difference of MT-ND6 and ANXA1 concentrations between septic survivor and non-survivor group

Among 134 patients with sepsis, the 30-day mortality rate was 26.9% (36/134 cases), more than one half of death occurred in the septic shock patients, and the mortality rate was 50.9% (27/53 cases). We compared the concentration of different parameters in septic patients according to the survival status. The results showed that only MT-ND6 exhibited significantly higher levels in non-surviving septic patients than surviving patients (**Figure 2D**). And no statistical difference was observed for the ANXA1, as well as the traditional clinical parameters PCT, CRP, IL-6 and HBP (**Figures 2H–L**).

### Predictive value of MT-ND6 and ANXA1 for 30-day mortality in adult patients with sepsis

**Figure 3:**
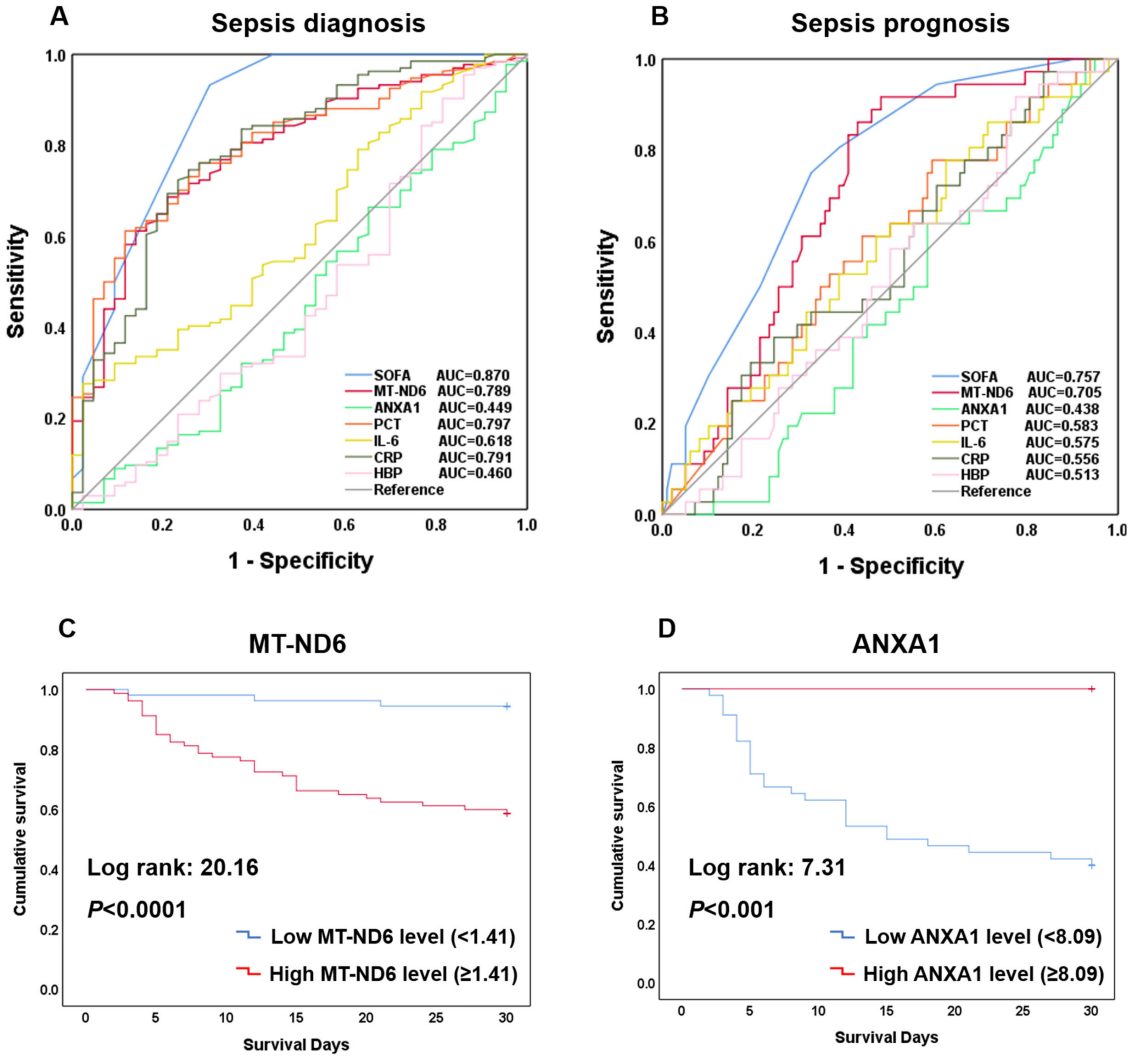
Efficacy of each indicators including SOFA, MT-ND6, ANXA1, PCT, IL-6, CRP, and HBP in diagnosing and prognosing patients with sepsis. **(A)** ROC curves of these indicators for diagnosing sepsis with ICU non-sepsis. **(B)** ROC curves of these indicators for prognosing the 30-day mortality. **(C)** Kaplan-Meier curve of MT-ND6 for 30-day survival. **(D)** Kaplan-Meier curve of ANXA1 for 30-day survival.

To investigate whether MT-ND6 and ANXA1 could be used as the diagnostic and prognostic biomarkers for the sepsis patients, ROC analysis and Kaplan-Meier survival curves was conducted. In terms of diagnosing sepsis patients with ICU non-sepsis patients, MT-ND6 showed an acceptable diagnostic value (AUC = 0.789) when compared with the PCT (AUC = 0.797) and CRP (AUC = 0.791), and the best AUC was observed for SOFA score (AUC = 0.870). However, ANXA1 did not exhibit any diagnostic efficiency in the discovery cohort (**Figure 3A**).

As for the prognostic values, same as we observed above, the AUC values of MT-ND6 ranked only second to SOFA score in predicting 30-day mortality of septic patients, which were 0.705 and 0.757 respectively (**Figure 3B**). Moreover, analyses of Kaplan-Meier survival curves revealed that patients with high serum MT-ND6 levels (≥ 1.41 ng/mL) displayed a lower survival rate than patients with low serum MT-ND6 levels (< 1.41 ng/mL) (*P* < 0.001) (**Figure 3C**).

Additionally, there was an unignorable trend that the concentration of ANXA1 seems to be lower in septic shock patients as well as those non-survivor patients, although there was no statistical difference. For further explore the potential prognostic value of ANXA1, we compared the ANXA1 levels in septic shock patients according to the survive status. The results showed that ANXA1 levels in septic shock non-survivor patients significantly decreased than those survivors (**Supplementary Figure S1A**). This seemed to portend that ANXA1 may be a protective molecule, whose decline was related to the high mortality in septic shock patients. Next, the ROC curve of ANXA1 (AUC = 0.665, *P* < 0.05) was conducted (**Supplementary Figure S1B**), with a cut-off value of 8.09 ng/mL, and the Kaplan-Meier survival curve was established (**Figure 3D**).

The AUC and the optimal research parameters cut-off points with their relevant validity indexes for diagnosing sepsis and predicting their 30-day mortality were shown in **Supplementary Table S1**.

### Validation of MT-ND6 and ANXA1 as a predictor of 30-day mortality

**Figure 4:**
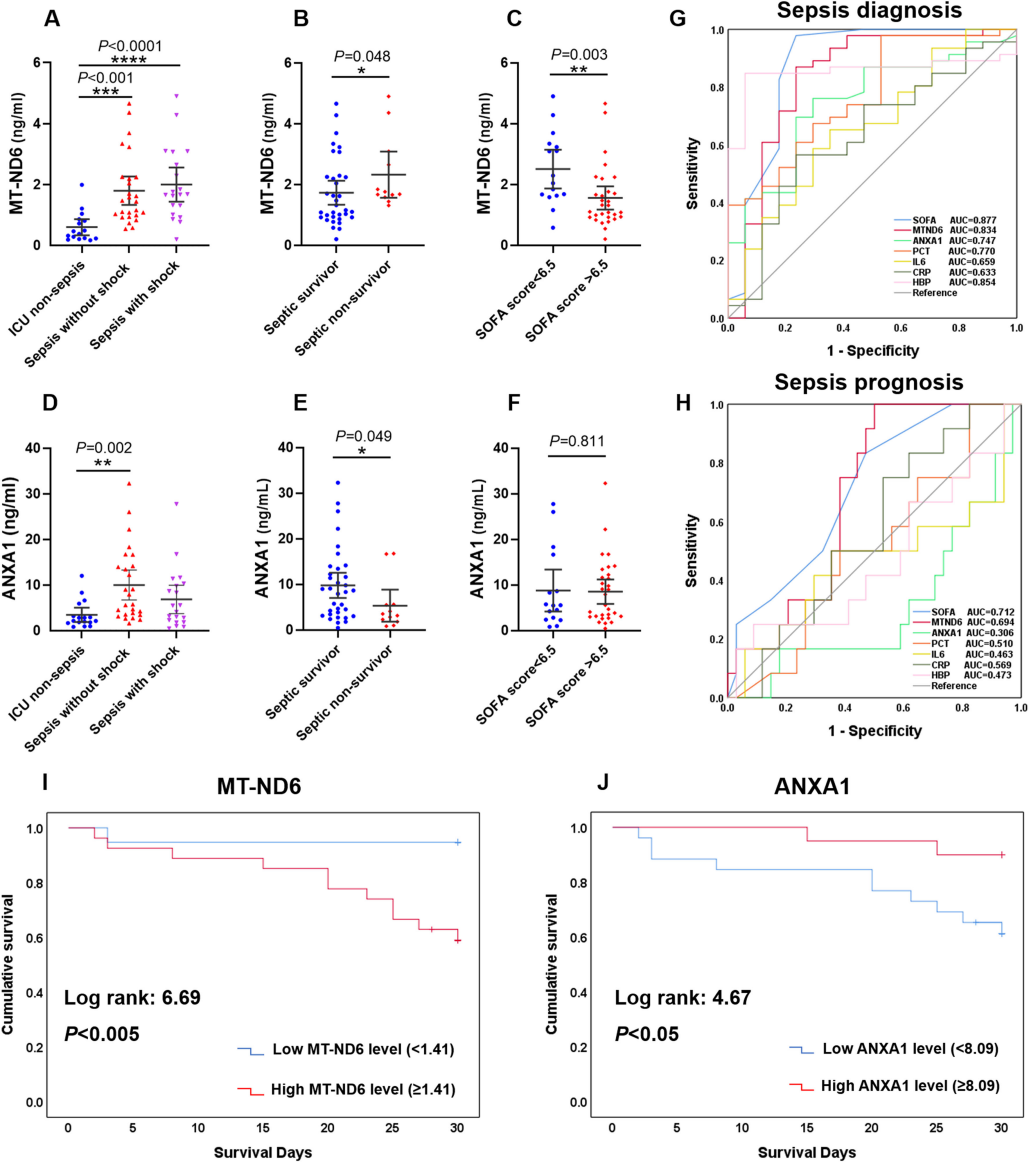
The serum levels of MT-ND6 and ANXA1 at admission were elevated in the validation cohort of patients with sepsis. **(A, D)** The serum concentrations of MT-ND6 and ANXA1 were detected from 26 sepsis patients without shock, 20 sepsis patients with shock and 17 ICU non-sepsis patients. MT-ND6 and ANXA1 concentrations were grouped by the survival states **(B, E)** and SOFA cut-off values **(C, F). (G, H)** The ROC curves of each indicators including SOFA, MT-ND6, ANXA1, PCT, IL-6, CRP, and HBP in diagnosing and prognosing sepsis patients in the validation cohort. **(I, J)** Kaplan-Meier curve of MT-ND6 and ANXA1 for 30-day survival in the validation cohort. The AUC values and confidence intervals, specificities and sensitivities of this tests are included in **Supplementary Table S1**. P ≤ 0.05 were considered statistically significant. ∗ denotes *P*< 0.05, ∗∗ denotes *P*< 0.01, ∗∗∗ denotes *P*< 0.001, ∗∗∗∗ denotes *P*< 0.0001 (Kruskal-Wallis and Mann–Whitney U test).

Similar to the findings above, the sepsis patients had significantly higher levels of MT-ND6 and ANXA1 compared with ICU non-sepsis patients in the validation cohort (**Figures 4A, D**). Patients suffering from death and with higher SOFA score values had a higher concentration of MT-ND6 (**Figures 4B, C**). And, the satisfactory diagnostic value (AUC = 0.834) and prognostic value (AUC = 0.694) of MT-ND6 displayed in the validation cohort implies that MT-ND61 may be a promising biomarker for sepsis (**Figures 4G, H**). In addition, septic non-survivor patients had lower serum ANXA1 levels than survivors (**Figure 4E**), but there was no statistic difference according to the SOFA scores (**Figure 4F**). Unlike with the rigorous performance in discovery cohort, ANXA1 exhibited satisfactory efficiencies for the validation cohort both in the diagnostic and prognostic aspects, with the AUC values were 0.747 and 0.694 respectively (**Figures 4G, H**). Kaplan-Meier survival curves for patients in the validation cohort according to the serum MT-ND6 and ANXA1 levels were computed. And the results showed that higher serum MT-ND6 levels (≥ 1.41 ng/mL) as well as lower serum ANXA1 levels (< 8.09 ng/mL) were respectively associated with higher probability of 30-day mortality in patients with sepsis (**Figures 4I, J**).

The AUC and the optimal research parameters cut-off points with their relevant validity indexes for diagnosing sepsis and predicting their 30-day mortality were shown in **Supplementary Table S2**.

### Criteria for immune status classification of sepsis patients by pro-inflammation and anti-inflammation cytokines

**Figure 5:**
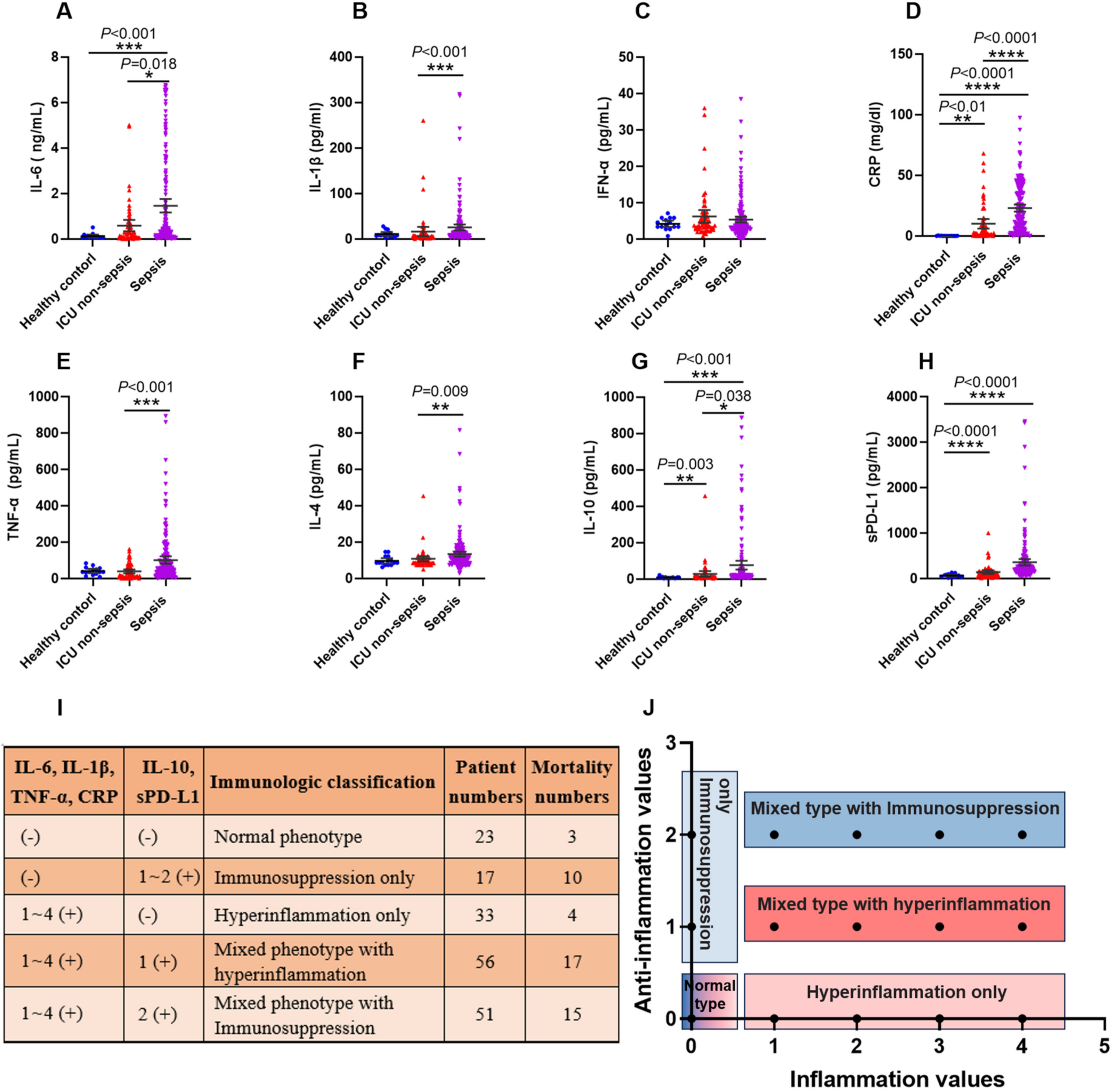
The criteria of immunological stratification according to the pro-inflammation and anti-inflammation cytokines in all of the sepsis patients. **(A–H)** The serum concentration of IL-6, IL-1β, IFN-α, CRP, TNF-α and IL-4, IL-10, sPD-L1 were detected from 180 sepsis patients, 43 ICU non-sepsis patients and 15 healthy controls. **(I, J)** The criteria for immune status classification of all sepsis patients according to these pro-inflammation and anti-inflammation cytokines, except for IFN-α and IL-4. *P* ≤ 0.05 were considered statistically significant. ∗ denotes *P*< 0.05, ∗∗ denotes *P*< 0.01, ∗∗∗ denotes *P*< 0.001, ∗∗∗∗ denotes *P*< 0.0001 (Kruskal-Wallis test).

Many cytokines, chemokines or other proteins have been studied as potential biomarkers to characterize a hyperinflammatory or immunosuppression state in sepsis patients. Here, we used five pro-inflammatory cytokines, namely interleukin-6 (IL-6), interleukin-1β (IL-1β), interferon-α (IFN-α), tumor necrosis factor-α (TNF-α), and CRP; and three anti-inflammation factors, namely interleukin-4 (IL-4), interleukin-10 (IL-10), and soluble programmed death ligand 1 (sPD-L1). The concentration of these cytokines in all of the subjects were shown in **Figures 5A–H**. We did not adopt IFN-α and IL-4 as the classifiers for the immune status of septic patients, because the levels of these two cytokines were below the detection threshold of the assay in a large number of patients.

We used 3 approaches to determine reference values for these cytokines, including using the 95th percentile reported by a clinical laboratory, the 95th percentile of the healthy volunteers and 50th percentile of the septic patients that detected in this study. The values varied across these approaches, and we used the highest value as reference for our study. Thus, the concentrations of these cytokines which higher than the reference values were marked as positive (+), otherwise marked as negative (-).

Next, we divided the septic patients into five phenotypes according to the positive numbers of the pro-inflammation and anti-inflammation cytokines (**Figures 5I, J**). The five phenotypes were normal immune status (n = 23), hyperinflammation only (n = 33), immunosuppression only (n = 17), mixed phenotypes with hyperinflammation (n = 56) and mixed phenotypes with immunosuppression (n = 51). The highest mortality was observed in the immunosuppression only phenotype, with 10 of 17 patients died within 30 days after ICU admission. The normal phenotype and hyperinflammation only phenotype delivered the lowest mortality, with 3 of 23 and 4 of 33 patients appeared poor outcomes respectively.

### MT-ND6 combined with ANXA1 improved the predictive values of 30-day mortality in patients with mixed inflammation

**Figure 6:**
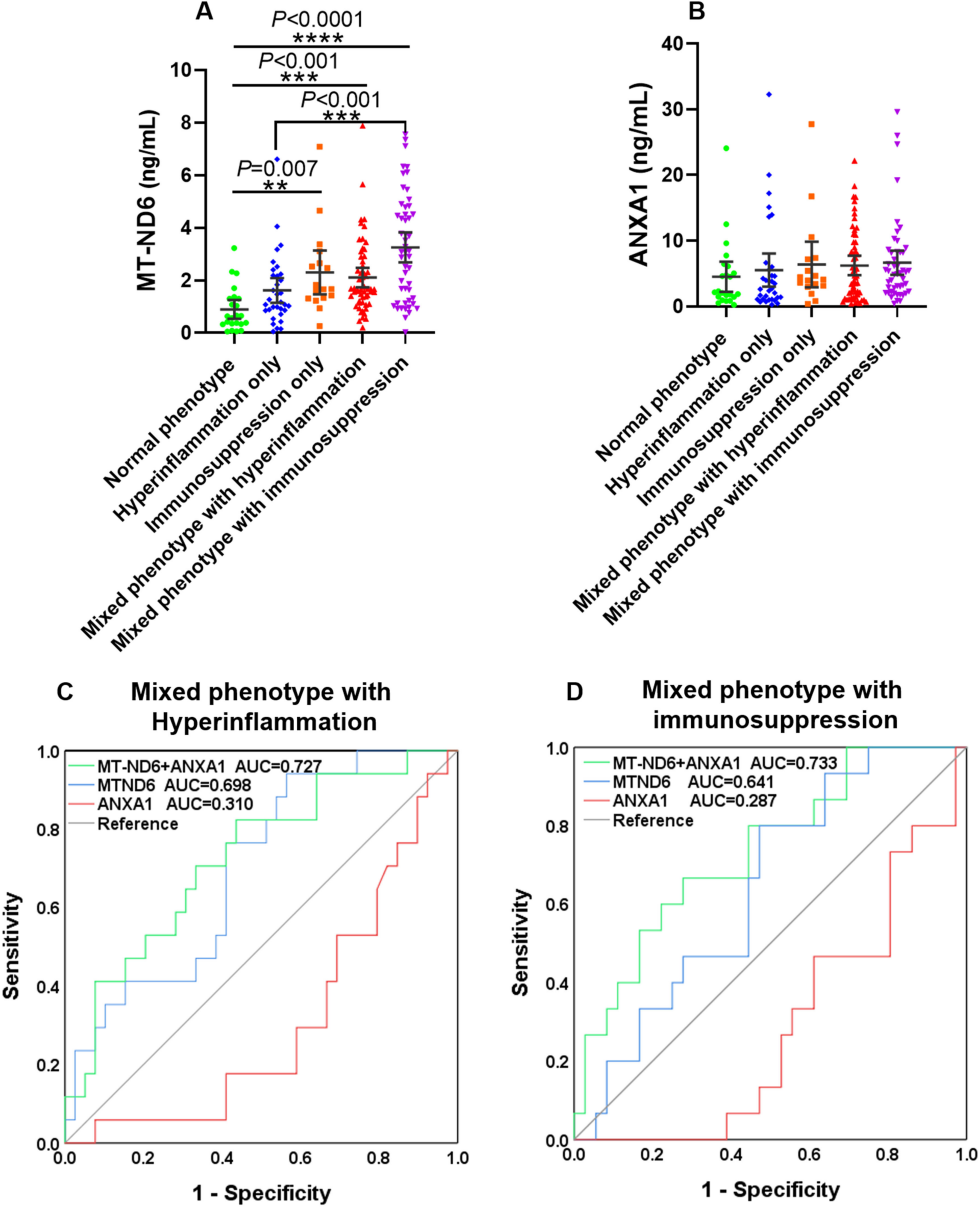
MT-ND6 combined with ANXA1 improved the predictive values of 30-day mortality in sepsis patients with mixed inflammatory phenotypes. **(A, B)** The serum concentration of MT-ND6 and ANXA1 in sepsis patients with different inflammation phenotypes. **(C, D)** The ROC curves of MT-ND6 and ANXA1 alone or in combination for predicting the 30-day mortality of sepsis patients with mixed inflammatory phenotypes. *P* ≤ 0.05 were considered statistically significant. ∗∗ denotes *P*< 0.01, ∗∗∗ denotes *P*< 0.001, ∗∗∗∗ denotes *P*< 0.0001 (Kruskal-Wallis test).

In order to further investigate the prediction role of MT-ND6 and ANXA1 for 30-day mortality in septic patients with different immune status, we used them alone or combined to estimate the 30-day mortality in all of the septic patients. The concentration of MT-ND6 and ANXA1 in the five immune phenotypes were shown in **Figures 6A, B**. The results showed that the plasma levels of MT-ND6 was significantly elevated in the patients with immunosuppression only phenotype as well as the mixed inflammation phenotypes. The same phenomenon was not observed in the hyperinflammation only group, whose MT-ND6 levels were significantly decreased when compared with the mixed phenotype with immunosuppression group (**Figure 6A**). Unexpectedly, there had no statistically differences of ANXA1 levels that was observed between the five immune statuses (**Figure 6B**).

Next, ROC analysis was conducted for prediction the 30-day mortality of these five phenotypes patients respectively. The results showed that MT-ND6 and ANXA1 delivered acceptable accuracy in predicting 30-day mortality in the mixed phenotype with hyperinflammation patients, and the AUC values were 0.698 and 0.310, respectively (*P* < 0.05). And the predictive values increased further to 0.727 when these two biomarkers were combined (**Figure 6C**). Additionally, in the mixed phenotype with immunosuppression group, only ANXA1 exhibited predictive efficacy with the AUC values were 0.287 (*P* < 0.05), and the predictive values were markedly increased to 0.733 (*P* < 0.01) when ANXA1 combined with MT-ND6, although there was no statistically significance observed on MT-ND6 (AUC = 0.641, *P* = 0.116) (**Figure 6D**). However, we did not observe any predictive values about these two biomarkers in the hyperinflammation only as well as the immunosuppression only group (**Supplementary Figures S2A, B**).

## Discussion

In this study, we detected the plasma levels of MT-ND6 and ANXA1 on ICU admission for diagnosing sepsis patients and predicting their 30-day mortality. Our research showed that MT-ND6 and ANXA1 were more effective in the short-term prognosis of sepsis than commonly used laboratory markers like PCT, CRP, IL-6 and HBP. Additionally, the immunological stratification of sepsis patients according to the pro-inflammatory and anti-inflammatory cytokines showed that the concentration of MT-ND6 was significantly increased with the degree of inflammation aggravated. And the combination of MT-ND6 and ANXA1 markedly improved the predictive values of 30-day mortality in patients with mixed hyperinflammation or immunosuppression phenotypes.

At present, the Sequential Organ Failure Assessment (SOFA) and Acute Physiology and Chronic Health Evaluation II (APACHEII) scores are the most prevalently used evaluation indicators when assessing organ failure and identifying disease severity. However, SOFA and APACHEII scores require many parameters, and the standardization of different assessors is critical, thus limiting its utility for early clinical decision-making (33).

Sepsis is characterized by immunosuppression and excessive inflammation (8, 9). Whether it concerns hyperinflammation or immunosuppression, it appears impossible that a single marker can accurately direct the immunological classify, since biomarkers are often related to one or more pathophysiological mechanisms/pathways. Therefore, a panel of biomarkers may reflect the immune status of the sepsis patient more precisely (21).

In our study, we used a range of pro-inflammatory and anti-inflammatory cytokines that well known for a long time to classify the immune statues of all the patients with sepsis (9, 34, 35), and the results showed that the concentration of MT-ND6 was significantly elevated as the immunological reaction aggravated, especially in the patients with mixed immune phenotypes and the immunosuppression only phenotype. Recently, more and more researchers recognized that it is not excessive immune activation, but rather immunosuppression, also known as “sepsis-induced immunoparalysis”is the overriding immune dysfunction associated with high mortality (21, 36). Indeed, the highest mortality of sepsis patients were observed in the immunosuppression only group in our study, with 10 of 17 patients died, instead, only 4 of 33 patients died in the hyperinflammation only group, exhibiting the lowest mortality. MT-ND6 is one of the most critical members of mtDAMPs, whose releases are recognized as the key step to activate the host innate immune response against pathogens (14, 19). However, the binding of mtFP to FPR1 on the PMN membrane can desensitize FPR1 and other GPCRs through receptor internalization, thereby reducing the chemotaxis of PMN (18, 37). Circulating mtFP seems to contribute to subsequent infections and increased mortality in septic shock patients who survive the initial phases of malignant inflammation (37). In our study, elevated plasma levels of MT-ND6 were observed in septic patients at the time of ICU admission, and the level of MT-ND6 correlated positively with the severity of the patient’s disease. Moreover, the binary logistic regression analysis showed that MT-ND6 (B = 0.318, OR (Odds ratio) = 1.374, *P* < 0.005) was independent risk factor of 30-day mortality (**Supplementary Table S3**). Recently, Kwon et al. successfully removed mtFPs in septic shock patients plasma by using antibody cocktail (through combining protein A/Sepharose with antibodies specific for ND6, ND3, ND4, and ND5) and rescued PMN FPR1-mediated [Ca2+]_i_ flux and chemotaxis that had been suppressed by earlier NFPs exposure in *in vitro* model systems (38). Although there has no *in vivo* trials to be undertaken to assess whether NFPs elimination improves pathogen infection control, this paradigm definitely represents a significant advancement in infectious disease risk management.

As an endogenous ligand of FPR1, ANXA1 exhibited a weaker relevance with the patient’s conditions. Although the slightly elevated levels of ANXA1 was observed in the plasma of sepsis patients, but there was no significant difference in the increase degree between the sepsis and ICU non-sepsis groups. Fortunately, we did not give up to explore the potential roles of ANXA1 and captured its decreasing tendency in the septic shock patients when compared with mild sepsis patients (*P* < 0.05).

Excessive inflammatory responses are critical pathologies that contribute to sepsis induced organic damage and death. Targeting anti-inflammatory ANXA1 and its receptors have achieved satisfyingly protective effects in many inflammation diseases, including sepsis-induced acute kidney injury (SI-AKI) and endotoxin-induced cerebral inflammation (39, 40). Furthermore, in our previous studies, we found that LPS attack raised the level of ANXA1 expression in the mice brain and that ANXA1 deletion dramatically increased the mortality rate of mice (41). To confirm the protection roles of ANXA1 in this study, we conducted ROC curves and survival analysis in the septic shock patients. The results were in line with our expectations, that low levels of ANXA1 (< 8.09 ng/mL) was able to predict the 30-day mortality, and the binary logistic regression analysis verified that ANXA1 was an independent protective factor in sepsis (B = -0.098, OR = 0.835, *P* = 0.020) (**Supplementary Table S3**).

In the present study, we found that another potential role of ANXA1 was as a complement to the predictive value of MT-ND6 for the 30-day mortality in patients with mixed inflammation phenotypes. As the predictive values boosted enormously when these two biomarkers were used in combination, whether it concerns mixed phenotype with hyperinflammation or mixed phenotype with immunosuppression. This combination brings us to a new treatment strategy that eliminate mtFPs and/or supplement with ANXA1-mimetic peptides might be a useful attempt to improve the complex anti-infection situations, which may be beneficial to clinical pathogen infection control and reduce the mortality rate in severe septic patients.

Despite the findings, this study has some shortcomings. First, our study included patients only in Shaoguan, Guangdong Province, China. Thus, multiple centers are needed for further study. Second, there are many ligands of FPR1, including the HSP, lipoxin A4, cathepsin G and so on, we only detected the concentration of MT-ND6 and ANXA1 in this study, which was insufficient to revel the comprehensive functions of FPR1 in sepsis. Third, sepsis is a complex syndrome involving multiple organ failure, and characterized by highly variable causes, clinical manifestations, and treatment measures, thus, a single time point cannot completely reflect the disease course and prognosis of the patients. So, large-sample, multi-center, and multi-time point clinical studies are needed to further explore the roles of FPR1 and its ligands in sepsis.

## Conclusions

Our data demonstrated that the diagnostic and prognostic values of MT-ND6 for sepsis patients might more powerful than conventional indicators. ANXA1 is an independent protector for sepsis, whose decreasing was related to the elevated mortality in severe septic patients. In addition, the combined use of these two molecules could obviously enhance the predictive values of 30-day mortality for septic patients with mixed inflammation phenotypes.

## Data Availability

The original contributions presented in the study are included in the article/**Supplementary Material**. Further inquiries can be directed to the corresponding author.

## Supplementary material

Supplementary Figure S1 The serum concentration of ANXA1 was significantly decreased in the discovery cohort of septic shock patients compared with septic without shock patients. **(A)** The serum levels of ANXA1 were grouped by the sepsis patients whether in shock. **(B)** The ROC curve of ANXA1 for predicting 30-day mortality in septic shock patients. *P* ≤ 0.05 were considered statistically significant. ∗ denotes *P*< 0.05 (Mann–Whitney U test).

Supplementary Figure S2 The ROC curves of MT-ND6 and ANXA1 for predicting 30-day mortality in sepsis patients with hyperinflammation and immunosuppression phenotypes. **(A, B)** The ROC curves of MT-ND6 and ANXA1 for predicting 30-day mortality in sepsis patients with hyperinflammation (n=33) and immunosuppression (n=17) phenotypes.

Supplementary Table S1 The AUC and the optimal research parameters cut-off points with their relevant validity indexes in diagnosing and prognosing sepsis patients in the discovery cohort. Note: AUC -the area under the ROC curve; SE (%)-sensitivity%; SP (%)-specificity%.

Supplementary Table S2 The AUC and the optimal research parameters cut-off points with their relevant validity indexes of biomarkers in diagnosing and prognosing sepsis patients in the validation cohort. Note: AUC -the area under the ROC curve; SE (%)-sensitivity%; SP (%)-specificity%.

Supplementary Table S3 The independent predictors of MT-ND6 and ANXA1 for 30-day mortality. Note: According to the cut-off value (MT-ND6 = 1.41 ng/mL; ANXA1 = 8.09 ng/mL), we judge the concentration of MT-ND6 and ANXA1 as negative (=0) and positive (=1) and then performed binary logistic regression.
