# Supplementary figures and images for: Serum mitochondrial-encoded NADH dehydrogenase 6 and Annexin A1 as novel biomarkers for mortality prediction in critically ill patients with sepsis
Source: Front Immunol. 2024 Nov 14;15:1486322. doi: 10.3389/fimmu.2024.1486322 (PMC11602424; doi:10.3389/fimmu.2024.1486322)

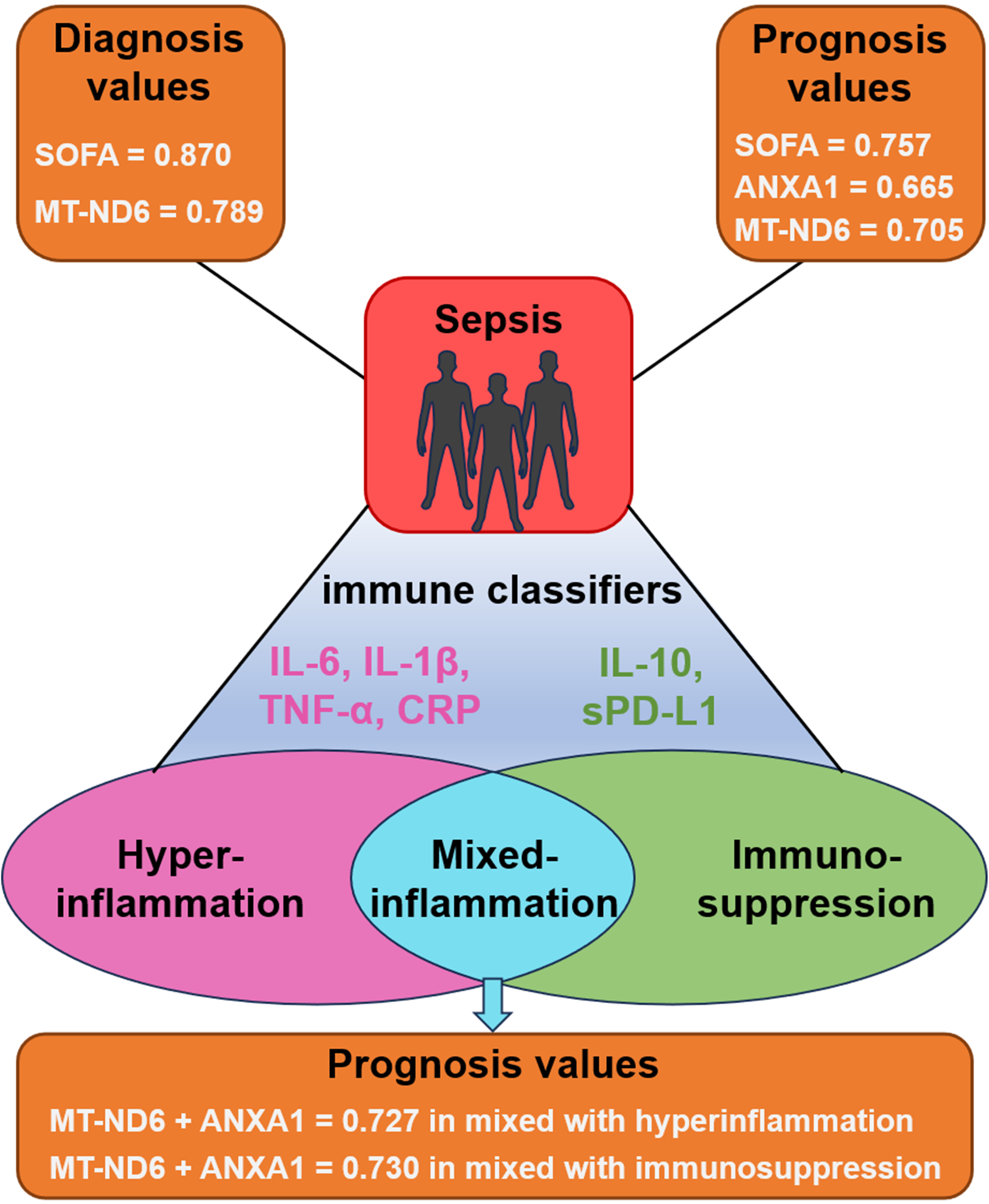

Supplement: Supplementary Figure S1 — The serum concentration of ANXA1 was significantly decreased in the discovery cohort of septic shock patients compared with septic without shock patients. (A) The serum levels of ANXA1 were grouped by the sepsis patients whether in shock. (B) The ROC curve of ANXA1 for predicting 30-day mortality in septic shock patients. P ≤ 0.05 were considered statistically significant. ∗ denotes P< 0.05 (Mann–Whitney U test). [file Image1.tif]

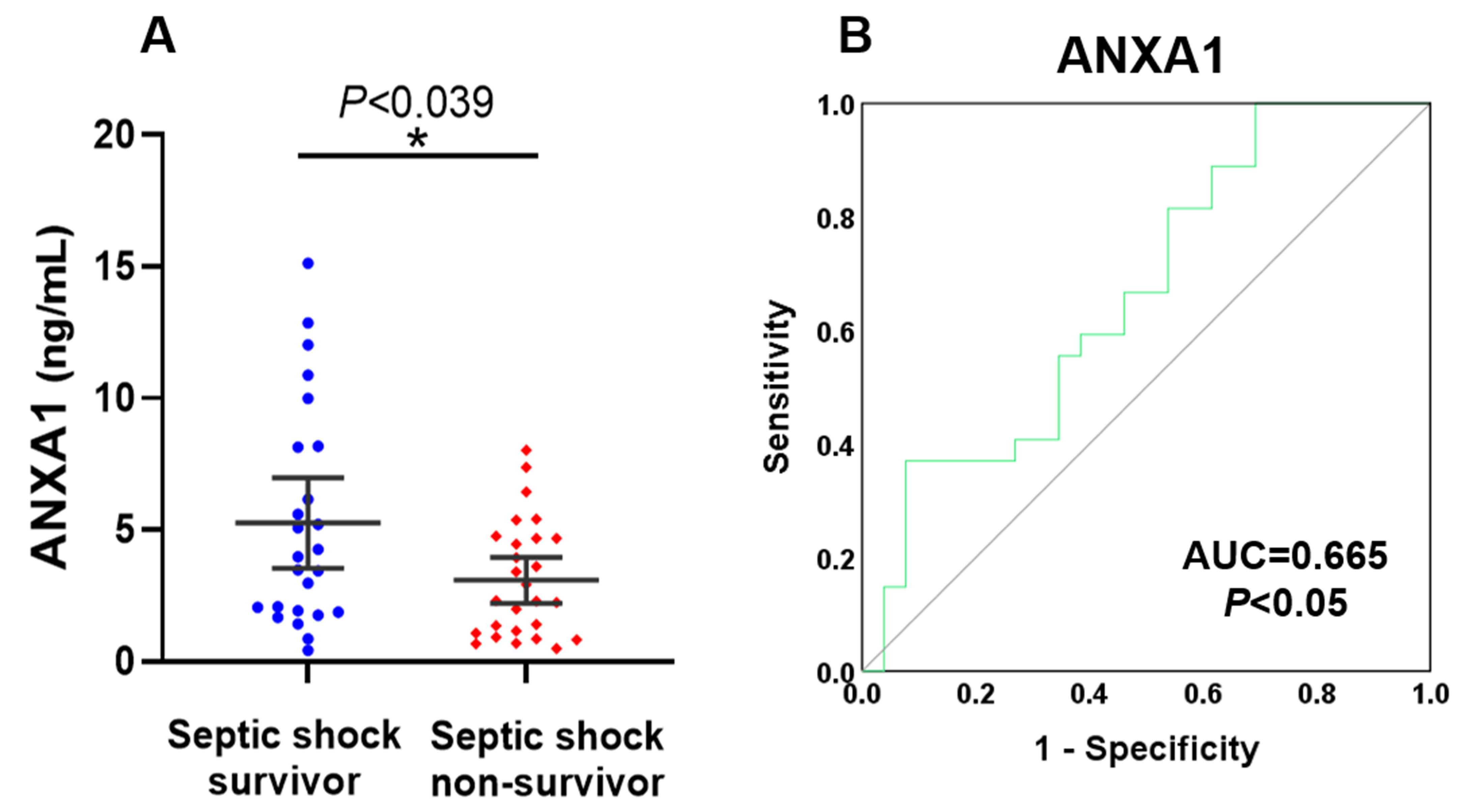

Supplement: Supplementary Figure S2 — The ROC curves of MT-ND6 and ANXA1 for predicting 30-day mortality in sepsis patients with hyperinflammation and immunosuppression phenotypes. (A, B) The ROC curves of MT-ND6 and ANXA1 for predicting 30-day mortality in sepsis patients with hyperinflammation (n=33) and immunosuppression (n=17) phenotypes. [file Image2.tif]

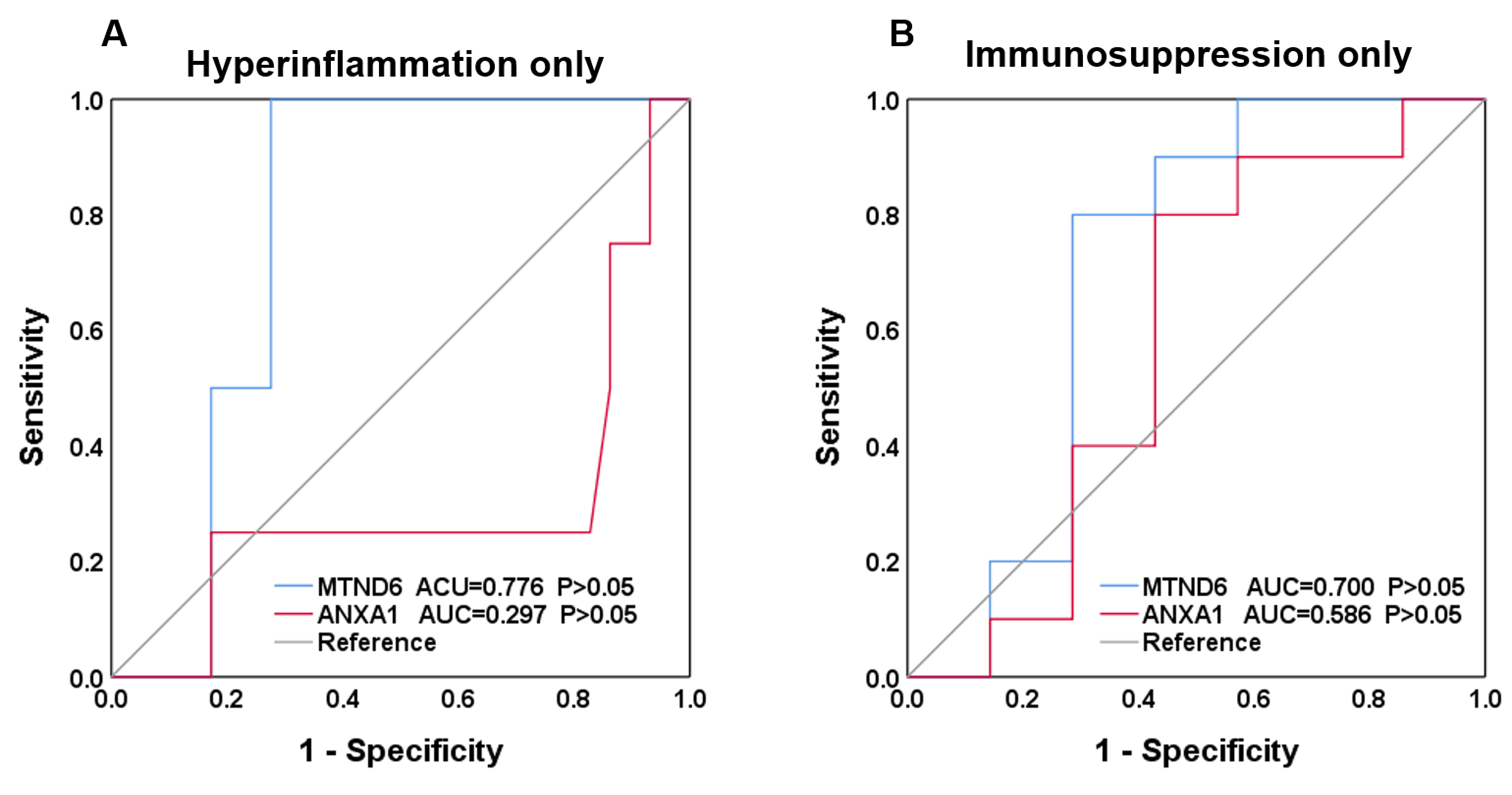

Supplement: Supplementary file 3 [file Image3.tif]
