# Supplementary material for: Serum mitochondrial-encoded NADH dehydrogenase 6 and Annexin A1 as novel biomarkers for mortality prediction in critically ill patients with sepsis
Source: Front Immunol. 2024 Nov 14;15:1486322. doi: 10.3389/fimmu.2024.1486322 (PMC11602424; doi:10.3389/fimmu.2024.1486322)
Supplement: Supplementary Table S1 — The AUC and the optimal research parameters cut-off points with their relevant validity indexes in diagnosing and prognosing sepsis patients in the discovery cohort. Note: AUC -the area under the ROC curve; SE (%)-sensitivity%; SP (%)-specificity%. [file Table1.docx]

| **Variables** | **Sepsis diagnosis** | | | | | |  | **Sepsis prognosis** | | | | | |
| --- | --- | --- | --- | --- | --- | --- | --- | --- | --- | --- | --- | --- | --- |
|  | **AUC** | **95% CI** | **Cut-off value** | **SE (%)** | **SP (%)** | ***P* value** |  | **AUC** | **95% CI** | **Cut-off value** | **SE (%)** | **SP (%)** | ***P* value** |
| SOFA | 0.870 | 0.800-0.941 | 3.5 | 93.3 | 69.8 | <0.001 | 0.757 | | 0.672-0.842 | 6.50 | 75.0 | 67.3 | <0.001 |
| MT-ND6 (ng/mL) | 0.789 | 0.714-0.863 | 1.16 | 68.7 | 79.1 | <0.001 | 0.705 | | 0.615-0.795 | 1.41 | 91.7 | 52.0 | <0.001 |
| ANXA1 (ng/mL) | 0.449 | 0.347-0.550 | 0.40 | 97.8 | 0.7 | 0.314 | 0.438 | | 0.335-0.541 | 2.24 | 63.9 | 41.8 | 0.273 |
| PCT (ng/mL) | 0.797 | 0.727-0.868 | 4.19 | 61.2 | 88.4 | <0.001 | 0.583 | | 0.477-0.689 | 3.22 | 77.8 | 40.8 | 0.141 |
| IL-6 (ng/mL) | 0.618 | 0.525-0.711 | 2.38 | 27.6 | 97.7 | <0.05 | 0.575 | | 0.467-0.684 | 0.18 | 77.8 | 37.8 | 0.182 |
| CRP (ng/dL) | 0.791 | 0.711-0.871 | 7.65 | 72.4 | 76.7 | <0.001 | 0.556 | | 0.450-0.662 | 35.47 | 38.9 | 75.5 | 0.320 |
| HBP (ng/mL) | 0.460 | 0.354-0.566 | 5.28 | 95.5 | 14.0 | 0.425 | 0.513 | | 0.408-0.617 | 10.99 | 91.7 | 22.4 | 0.819 |
