# Supplementary material for: Serum mitochondrial-encoded NADH dehydrogenase 6 and Annexin A1 as novel biomarkers for mortality prediction in critically ill patients with sepsis
Source: Front Immunol. 2024 Nov 14;15:1486322. doi: 10.3389/fimmu.2024.1486322 (PMC11602424; doi:10.3389/fimmu.2024.1486322)
Supplement: Supplementary Table S2 — The AUC and the optimal research parameters cut-off points with their relevant validity indexes of biomarkers in diagnosing and prognosing sepsis patients in the validation cohort. Note: AUC -the area under the ROC curve; SE (%)-sensitivity%; SP (%)-specificity%. [file Table2.docx]

| **Variables** | **Sepsis diagnosis** | | | | | |  | **Sepsis prognosis** | | | | | |
| --- | --- | --- | --- | --- | --- | --- | --- | --- | --- | --- | --- | --- | --- |
|  | **AUC** | **95% CI** | **Cut-off value** | **SE (%)** | **SP (%)** | ***P* value** |  | **AUC** | **95% CI** | **Cut-off value** | **SE (%)** | **SP (%)** | ***P* value** |
| SOFA | 0.875 | 0.747-1.000 | 3.5 | 82.6 | 82.4 | <0.001 | 0.728 | | 0.575-0.881 | 6.50 | 50.0 | 70.6 | <0.05 |
| MT-ND6 (ng/mL) | 0.836 | 0.701-0.971 | 1.16 | 63.0 | 82.4 | <0.001 | 0.694 | | 0.542-0.845 | 1.41 | 91.7 | 52.9 | <0.05 |
| ANXA1 (ng/mL) | 0.751 | 0.621-0.880 | 0.40 | 97.8 | 0.0 | <0.005 | 0.306 | | 0.129-0.484 | 2.24 | 66.7 | 8.8 | <0.05 |
| PCT (ng/mL) | 0.763 | 0.636-0.891 | 4.19 | 71.7 | 52.9 | <0.005 | 0.510 | | 0.329-0.691 | 3.22 | 75.0 | 26.5 | 0.920 |
| IL-6 (ng/mL) | 0.638 | 0.483-0.793 | 2.38 | 15.2 | 94.1 | 0.094 | 0.463 | | 0.247-0.679 | 0.18 | 58.3 | 20.6 | 0.707 |
| CRP (ng/dL) | 0.616 | 0.461-0.772 | 7.65 | 71.7 | 41.2 | 0.159 | 0.569 | | 0.395-0.742 | 35.47 | 25.0 | 73.5 | 0.484 |
| HBP (ng/mL) | 0.838 | 0.737-0.939 | 5.28 | 84.8 | 35.3 | <0.001 | 0.473 | | 0.270-0.676 | 10.99 | 41.7 | 47.1 | 0.783 |
