# Supplementary material for: Serum mitochondrial-encoded NADH dehydrogenase 6 and Annexin A1 as novel biomarkers for mortality prediction in critically ill patients with sepsis
Source: Front Immunol. 2024 Nov 14;15:1486322. doi: 10.3389/fimmu.2024.1486322 (PMC11602424; doi:10.3389/fimmu.2024.1486322)
Supplement: Supplementary Table S3 — The independent predictors of MT-ND6 and ANXA1 for 30-day mortality. Note: According to the cut-off value (MT-ND6 = 1.41 ng/mL; ANXA1 = 8.09 ng/mL), we judge the concentration of MT-ND6 and ANXA1 as negative (=0) and positive (=1) and then performed binary logistic regression. [file Table3.docx]

| **Parameter** | **B** | **Stand error** | **WALD** | **Degrees of freedom** | ***P* value** | **Odds ratio** | **95% CI** |
| --- | --- | --- | --- | --- | --- | --- | --- |
| Age | 0.036 | 0.016 | 4.976 | 1 | 0.026 | 1.037 | 1.004-1.070 |
| MT-ND6 | 0.318 | 0.101 | 9.897 | 1 | 0.002 | 1.374 | 1.127-1.675 |
| ANXA1 | -0.098 | 0.042 | 5.438 | 1 | 0.020 | 0.835 | 0.835-0.984 |
| Lymphocyte | -0.066 | 0.284 | 0.054 | 1 | 0.817 | 0.936 | 0.537-1.634 |
